# Statin treatment alters serum n-3 to n-6 polyunsaturated fatty acids ratio in patients with dyslipidemia

**DOI:** 10.1186/s12944-015-0066-6

**Published:** 2015-07-07

**Authors:** Tsuyoshi Nozue, Ichiro Michishita

**Affiliations:** Division of Cardiology, Department of Internal Medicine, Yokohama Sakae Kyosai Hospital, Federation of National Public Service Personnel Mutual Associations, 132 Katsura-cho, Sakae-ku, Yokohama, 247-8581 Japan

**Keywords:** Docosahexaenoic acid, Eicosapentaenoic acid, Statin, Polyunsaturated fatty acids

## Abstract

**Background:**

The effects of statins on serum n-3 to n-6 polyunsaturated fatty acids (PUFAs) levels have not been fully evaluated. We examined the effects of two types of statins (rosuvastatin and pitavastatin) on serum PUFAs levels and their ratios in patients with dyslipidemia.

**Findings:**

A total of 46 patients who were not receiving lipid-lowering therapy were randomly assigned to receive either 2.5 mg/day of rosuvastatin or 2 mg/day of pitavastatin. Serum PUFAs levels were measured at baseline, at 4 weeks, and at 12 weeks. Rosuvastatin was used to treat 23 patients, and the remaining 23 patients were treated using pitavastatin. Serum docosahexaenoic acid (DHA) levels decreased significantly at 12 weeks in both groups (rosuvastatin: from 169.6 to 136.3 μg/mL, p = 0.006; pitavastatin: from 188.6 to 153.9 μg/mL, p = 0.03). However, serum levels of eicosapentaenoic acid (EPA) and arachidonic acid (AA) did not change. In addition, the EPA/AA ratio did not change, whereas the DHA/AA ratio decreased significantly at 12 weeks in both groups (rosuvastatin: from 0.99 to 0.80, p = 0.01; pitavastatin: from 1.14 to 0.91, p = 0.003). No adverse events were observed during the study period.

**Conclusions:**

In this small, open-label, pilot study, rosuvastatin and pitavastatin decreased serum DHA levels and the DHA/AA ratio in patients with dyslipidemia.

## Background

Numerous clinical trials have shown that statins can significantly reduce the incidence of cardiovascular disease. Furthermore, intensive lipid-lowering therapy with statins yields a significantly greater reduction in the risk of coronary events compared to moderate lipid-lowering therapy [1, 2]. Nonetheless, a residual risk is present in all of the statin trials [3]. Statins and polyunsaturated fatty acids (PUFAs) have similar actions such as enhance endothelial nitric synthesis, inhibit the production of pro-inflammatory cytokines, and prevent atherosclerosis [4]. Thus, the residual risk of cardiovascular events after statin therapy is explained in part by the n-3 to n-6 polyunsaturated fatty acids (PUFAs) ratios [5].

The effects of statins on serum n-3 to n-6 PUFAs levels and these ratios have not been fully evaluated. Recently, we reported that pitavastatin decreased serum docosahexaenoic acid (DHA)/arachidonic acid (AA) ratio, whereas pravastatin had no effect on this ratio [6]. However, Nakamura et al. reported that pravastatin and simvastatin decreased serum eicosapentaenoic acid (EPA)/AA ratio [7]. Harris et al. also reported that serum EPA/AA ratio was decreased by simvastatin [8]. Based on these reports, we hypothesized that the effects of statin on n-3 to n-6 PUFAs ratios would be depend on types of statin. To test this hypothesis, we examined the effects of two types of statins (rosuvastatin and pitavastatin) on serum PUFAs levels and their ratios in patients with dyslipidemia.

## Methods

### Patients and study design

Between June 2009 and May 2011, outpatients with dyslipidemia who were being treated at our hospital were enrolled in this study if they had not achieved the target lipid levels recommended by the Japan Atherosclerosis Society Guidelines [9] despite diet and exercise for 3 months. The following patients were excluded from the study: (1) those treated with lipid-lowering drugs (statins, PUFAs, fibrates, nicotinic acid, colestyramine, or probucol), (2) those with dyslipidemia associated with hypothyroidism, nephrotic syndrome, gallbladder obstruction, biliary disease, pancreatitis, or immunologic abnormalities such as collagen diseases, (3) those with dyslipidemia induced by steroids or other drugs, (4) those with severe liver dysfunction, (5) alcoholism or heavy alcohol intake, (6) any other reason for which the patient was considered inappropriate for this study by the attending physician.

A total of 46 patients were randomly assigned to receive either 2.5 mg/day of rosuvastatin or 2 mg/day of pitavastatin. Serum PUFAs levels were measured at baseline, at 4 weeks, and at 12 weeks. Adverse effects were assessed at each study visit by patient interviews and laboratory testing. During the study period, there were no lifestyle changes, the use of non-study anti-dyslipidemic agents was prohibited, and anti-hypertensive and anti-diabetic therapy used at enrollment was continued without dosage modifications.

There are 2 distinct classes of statins: lipophilic (pitavastatin, atorvastatin, fluvastatin, and simvastatin) and hydrophilic (pravastatin and rosuvastatin) [10]. Pitavastatin is a commonly used lipophilic strong statin in Japan, and its ability to lower serum low-density lipoprotein cholesterol (LDL-C) is comparable with that of atorvastatin [11]. Rosuvastatin was selected as hydrophilic strong statin.

This study was conducted in accordance with the Declaration of Helsinki and with the approval of the ethical committees of Yokohama Sakae Kyosai Hospital. Written informed consent was obtained from each patient enrolled in the study.

### Laboratory determinations

Blood samples were obtained after an overnight fast at baseline, at 4 weeks, and at 12 weeks. Serum levels of total cholesterol, LDL-C, high-density lipoprotein cholesterol (HDL-C), and triglycerides (TG) were measured by standard enzymatic methods on AU2700 (Beckman Coulter, CA, USA) using commercial enzymatic kits (Kyowa Medex, Tokyo, Japan). Serum apolipoprotein A1, B, and E levels were measured by turbidimetric immunoassay at a central laboratory (SRL Inc., Tokyo, Japan). Serum levels of four bioactive fatty acids, including EPA, DHA, AA, and dihomogamma-linolenic acid, were measured using a gas chromatograph (GC-2010; Shimadzu Corporation, Kyoto, Japan) equipped with a capillary column (TC-70; GL Sciences, Tokyo, Japan) at a central laboratory (SRL Inc., Tokyo, Japan).

### Statistical analysis

Statistical analyses were performed using StatView version 5.0 (SAS Institute, Cary, North Carolina, USA). The results were expressed as median (range). The Wilcoxon signed rank test was used to compare the data before and after statin therapy. Categorical variables between the 2 groups were compared using the chi-square test or the Fisher’s exact test. The statistical significance level was set at p < 0.05. Corrections for multiple comparisons and a sample-size calculation were not performed.

## Findings

Twenty-three patients were assigned to rosuvastatin group, and the remaining 23 patients to pitavastatin group. The baseline characteristics of subjects are shown in Table 1. The baseline characteristics did not differ between the 2 groups. The number of diabetes mellitus was 9 in rosuvastatin group and 11 in pitavastatin group. Five patients in rosuvastatin group and 4 patients in pitavastatin group had a prior history of coronary artery disease.

**Table 1:** Baseline characteristics of subjects

	Rosuvastatin (n = 23)	Pitavastatin (n = 23)	p value
Age (years)	69 (48-86)	66 (47-83)	0.31
Male gender, n	12/23	14/23	0.55
Height (cm)	163 (145-173)	163 (147-180)	0.8
Body weight (kg)	63 (44-88)	65 (38-80)	0.35
BMI (kg/m^2^)	23.5 (17.6-32.6)	24.0 (17.3-28.7)	0.5
Smoking, n	2/23	2/23	>0.99
History of CAD, n	5/23	4/23	>0.99
Diabetes mellitus, n	9/23	11/23	0.55
Hypertension, n	14/23	9/23	0.14

Data are expressed as median (range) or number

BMI, body mass index; CAD, coronary artery disease

The effects of statins on lipid levels are shown in Table 2. Serum levels of total cholesterol, LDL-C, TG, apolipoprotein B and E decreased significantly in both groups. However, significant increases in HDL-C and apolipoprotein A1 levels were observed only in rosuvastatin group.

**Table 2:** Effects of rosuvastatin and pitavastatin on serum lipid levels

		Baseline	4 weeks	p value	12 weeks	p value
TC (mg/dL)	Rosuvastatin	232 (160-288)	163 (126-209)	<0.0001	171 (125-206)	<0.0001
	Pitavastatin	249 (174-301)	178 (111-215)	<0.0001	183 (118-252)	<0.0001
LDL-C (mg/dL)	Rosuvastatin	159 (103-216)	83 (55-145)	<0.0001	88 (50-138)	<0.0001
	Pitavastatin	176 (117-240)	106 (46-155)	<0.0001	98 (48-180)	<0.0001
HDL-C (mg/dL)	Rosuvastatin	56 (36-79)	61 (40-90)	0.07	63 (38-103)	0.005
	Pitavastatin	60 (41-93)	62 (41-125)	0.14	63 (40-120)	0.16
TG (mg/dL)	Rosuvastatin	164 (75-396)	116 (70-211)	0.002	110 (71-312)	0.009
	Pitavastatin	169 (84-303)	134 (61-316)	0.01	121 (64-311)	0.01
Apo Al (mg/dL)	Rosuvastatin	144 (106-172)	151 (116-192)	0.01	147 (116-234)	0.004
	Pitavastatin	150 (119-203)	149 (100-256)	0.35	152 (94-257)	0.19
Apo B (mg/dL)	Rosuvastatin	116 (76-159)	75 (52-106)	<0.0001	77 (49-103)	<0.0001
	Pitavastatin	132 (86-176)	87 (43-111)	<0.0001	88 (43-143)	<0.0001
Apo E (mg/dL)	Rosuvastatin	4.7 (3.6-7.6)	3.7 (2.6-5.2)	<0.0001	3.6 (2.4-4.6)	<0.0001
	Pitavastatin	5.4 (3.3-11.9)	4.3 (2.2-8.0)	<0.0001	4.3 (2.7-9.7)	<0.0001

Data are expressed as median (range)

TC, total cholesterol; LDL-C, low-density lipoprotein cholesterol; HDL-C, high-density lipoprotein cholesterol; TG, trigycerides; Apo, apolipoprotein

Serum PUFAs levels and their ratios at baseline, at 4 weeks, and at 12 weeks are shown in Table 3. Serum levels of EPA and AA did not change, whereas the DHA levels decreased significantly at 12 weeks in both groups (rosuvastatin: from 169.6 to 136.3 μg/mL, p = 0.006; pitavastatin: from 188.6 to 153.9 μg/mL, p = 0.03). As a result, a significant decrease in the DHA/AA ratio was observed (rosuvastatin: from 0.99 to 0.80, p = 0.01; pitavastatin: from 1.14 to 0.91, p = 0.003), whereas the EPA/AA ratio showed no significant change in both groups. No adverse events, including the occurrence of clinical symptoms or changes in the biochemical parameters, were observed during the study period.

**Table 3:** Effects of rosuvastatin and pitavastatin on serum PUFAs levels and their ratios

		Baseline	4 weeks	p value	12 weeks	p value
EPA (*μ*g/mL)	Rosuvastatin	66.9 (28.9-199.9)	70.2 (31.5-156.7)	0.69	64. 8 (23.7-216.9)	0.83
	Pitavastatin	87.0 (15.2-149.8)	65.6 (16.0-201.5)	0.06	79.8 (17.8-205.0)	0.51
DHA (*μ*g/mL)	Rosuvastatin	169.6 (98.1-384-6)	142.5 (66.0-215.6)	0.005	136.3 (56.0-251.9)	0.006
	Pitavastatin	188.6 (91.8-283.6)	135.2 (80.3-198.8)	0.0004	153.9 (93.8-250.4)	0.03
DGLA (*μ*g/mL)	Rosuvastatin	36.2 (18.1-74.4)	34.2 (18.3-53.3)	0.06	33.6 (17.1-56.5)	0.18
	Pitavastatin	37.4 (18.4-57.3)	35.4 (20.0-53.5)	0.13	36.7 (23.5-58.9)	0.48
AA (*μ*g/mL)	Rosuvastatin	168.3 (114.6-284.9)	158.3 (104.2-276.7)	0.43	163.2 (88.9-269.2)	0.81
	Pitavastatin	167.3 (106.0-279.2)	166.0 (102.0-242.6)	0.89	180.2 (97.0-236.9)	0.2
EPA/AA	Rosuvastatin	0.42 (0.13-1.65)	0.44 (0.13-1.45)	0.36	0.37 (0.10-1.48)	0.91
	Pitavastatin	0.51 (0.07-1.19)	0.38 (0.07-1.27)	0.14	0.43 (0.08-1.18)	0.25
DHA/AA	Rosuvastatin	0.99 (0.42-1.82)	0.81 (0.37-1.69)	0.05	0.80 (0.35-1.76)	0.01
	Pitavastatin	1.14 (0.08-1.18)	0.87 (0.37-1.77)	0.0002	0.91 (0.45-1.59)	0.003
EPA + DHA/AA	Rosuvastatin	1.42 (0.55-3.34)	1.25 (0.50-3.14)	0.54	1.25 (0.45-2.88)	0.07
	Pitavastatin	1.75 (0.52-2.85)	1.22 (0.44-2.89)	0.002	1.34 (0.53-2.64)	0.03

Data are expressed as median (range)

PUFAs, polyunsaturated fatty acids; EPA, eicosapentaenoic acid; DHA, docosahexaenoic acid; DGLA, dihomogamma-linolenic acid; AA, arachidonic acid

## Discussion

The major findings of the present study are as follows: (1) rosuvastatin and pitavastatin decreased serum DHA levels and a significant decrease in the DHA/AA ratio was observed, and (2) the EPA/AA ratio showed no significant change in both statins.

Few studies have reported the effects of statins on n-3 to n-6 PUFA ratios. One study reported that pravastatin and simvastatin increased the AA level significantly whereas they did not affect EPA and DHA levels, resulting in a significant decrease in the EPA/AA ratio [7]. The other study reported that concentration of AA were unchanged while the EPA/AA ratio was decreased significantly by simvastatin [8]. On the other hand, rosuvastatin, pitavastatin, and atorvastatin mainly reduced n-3 PUFAs [12]. According to these previous reports, the effects of statins on serum PUFAs levels have varied. However, we reported recently that pitavastatain decreased the DHA/AA ratio, whereas pravastatin had no effect on this ratio [6]. Thus, considering of the results of this study, it seems that strong statins (e.g., rosuvastatin, pitavastatin, atorvastatin) mainly affect serum DHA level, and the DHA/AA ratio would be decreased by these types of statin. On the other hand, first generation weak statins (e.g., pravastatin, simvastatin) mainly decrease the EPA/AA ratio.

The mechanisms of how statin treatment affects serum PUFAs level have not been fully examined. Jula et al. reported that simvastatin increases serum AA levels due to an increase in Δ6- and Δ5-desaturase enzyme activities [13]. Although the effects of other statins on these enzyme activities have not been evaluated, statins increase the formation of AA, EPA, and DHA by these enzymes [14]. The AA, EPA, and DHA give rise to anti-inflammatory molecules such as lipoxins, resolvins, and protectins [14]. Some of beneficial actions of statins are explained by the formation of these anti-inflammatory molecules [15]. Thus, PUFAs and their metabolites may serve as second messengers of the actions of statins [4]. We speculate that the effects of statins on the formation and conversion of PUFAs may be different among specific types of statins.

In the JELIS trial, EPA added on statins (10 mg of pravastatin or 5 mg of simvastatin) significantly decreased the incidence of coronary events by 19 % [16]. Thus, combination of EPA with statins has been demonstrated to reduce future cardiovascular events for primary and secondary prevention [17, 18]. Statins as well as PUFAs can inhibit HMG-CoA reductase activity [19]. Inhibition of the mevalonate pathway by statins and PUFAs prevents the function of small guanosine triphosphatases and enhance the expression of bone morphogenetic proteins [20]. In view of the similarity in their actions, combination PUFAs with statins may prove to be highly beneficial clinical effects. We consider that DHA add on strong statins may be useful strategy to prevent cardiovascular events, because strong statins mainly decrease the DHA/AA ratio.

The present study had several limitations. First, this was a small, open-label, pilot study and the observation period was only 12 weeks. Second, we did not evaluate total fatty acids. Third, lifestyle habits were not controlled, and changes in the PUFAs composition of the diet are reflected in the levels of serum PUFAs. In addition, exercise or smoking may have affected the present results. Therefore, a further, adequately powered, randomized, controlled trial is necessary to confirm the effect of different types of statin on n-3 to n-6 PUFAs ratios.

## Conclusions

In this small, open-label, pilot study, rosuvastatin and pitavastatin decreased serum DHA levels and the DHA/AA ratio in patients with dyslipidemia.

## Abbreviations

AA: Arachidonic acid
DHA: Docosahexaenoic acid
EPA: Eicosapentaenoic acid
HDL-C: High-density lipoprotein cholesterol
JELIS: Japan EPA lipid intervention study
LDL-C: Low-density lipoprotein cholesterol
PUFAs: Polyunsaturated fatty acids
TG: Triglycerides
